# LAT1 Protein Content Increases Following 12 Weeks of Resistance Exercise Training in Human Skeletal Muscle

**DOI:** 10.3389/fnut.2020.628405

**Published:** 2021-01-14

**Authors:** Paul A. Roberson, C. Brooks Mobley, Matthew A. Romero, Cody T. Haun, Shelby C. Osburn, Petey W. Mumford, Christopher G. Vann, Rory A. Greer, Arny A. Ferrando, Michael D. Roberts

**Affiliations:** ^1^School of Kinesiology, Auburn University, Auburn, AL, United States; ^2^Department of Physiology, College of Medicine, University of Kentucky, Lexington, KY, United States; ^3^Department of Geriatrics, Donald W. Reynolds Institute on Aging, University of Arkansas for Medical Sciences, Little Rock, AK, United States

**Keywords:** amino acid metabolism, ATF4, protein supplementation, protein synthesis, BCKDH

## Abstract

**Introduction:** Amino acid transporters are essential for cellular amino acid transport and promoting protein synthesis. While previous literature has demonstrated the association of amino acid transporters and protein synthesis following acute resistance exercise and amino acid supplementation, the chronic effect of resistance exercise and supplementation on amino acid transporters is unknown. The purpose herein was to determine if amino acid transporters and amino acid metabolic enzymes were related to skeletal muscle hypertrophy following resistance exercise training with different nutritional supplementation strategies.

**Methods:** 43 college-aged males were separated into a maltodextrin placebo (PLA, *n* = 12), leucine (LEU, *n* = 14), or whey protein concentrate (WPC, *n* = 17) group and underwent 12 weeks of total-body resistance exercise training. Each group's supplement was standardized for total energy and fat, and LEU and WPC supplements were standardized for total leucine (6 g/d). Skeletal muscle biopsies were obtained prior to training and ~72 h following each subject's last training session.

**Results:** All groups increased type I and II fiber cross-sectional area (fCSA) following training (*p* < 0.050). LAT1 protein increased following training (*p* < 0.001) and increased more in PLA than LEU and WPC (*p* < 0.050). BCKDHα protein increased and ATF4 protein decreased following training (*p* < 0.001). Immunohistochemistry indicated total LAT1/fiber, but not membrane LAT1/fiber, increased with training (*p* = 0.003). Utilizing all groups, the change in ATF4 protein, but no other marker, trended to correlate with the change in fCSA (*r* = 0.314; *p* = 0.055); however, when regression analysis was used to delineate groups, the change in ATF4 protein best predicted the change in fCSA only in LEU (*r*^2^ = 0.322; *p* = 0.043). In C2C12 myoblasts, LAT1 protein overexpression caused a paradoxical decrease in protein synthesis levels (*p* = 0.002) and decrease in BCKDHα protein (*p* = 0.001).

**Conclusions:** Amino acid transporters and metabolic enzymes are affected by resistance exercise training, but do not appear to dictate muscle fiber hypertrophy. In fact, overexpression of LAT1 *in vitro* decreased protein synthesis.

## Introduction

Skeletal muscle possesses the unique ability to adapt to metabolic and loading demands. For example, disuse and physical inactivity contribute to muscle atrophy (1, 2), while both aerobic and resistance training can improve skeletal muscle quality and promote hypertrophy (3, 4). To enhance exercise training adaptations it has been suggested to ingest upwards of 1.6–2.0 g/kg of dietary protein (5–7). While it remains to be fully determined how greater consumption of amino acids promotes muscle growth, essential amino acids (and leucine in particular) are capable of simulating increases in muscle protein synthesis suggesting a positive role for amino acids in promoting skeletal muscle hypertrophy (8–10).

In skeletal muscle, amino acid transporters function by transporting amino acids that are unique to specific transporters from the interstitial fluid to the sarcoplasm of muscle fibers. For instance, the L-type amino acid transporter 1 (LAT1) primarily transports leucine into the sarcoplasm while co-transporting glutamine out of the cell, whereas the sodium-coupled neutral amino acid transporter 2 (SNAT2) co-transports both sodium and glutamine into the sarcoplasm. These two transport proteins work in tandem such that SNAT2 brings in glutamine for LAT1 to pump back out of the cell while concomitantly transporting leucine into the cell. Leucine has been deemed as an essential regulator of protein synthesis (11), and independently increases protein synthesis through activation of the mechanistic target of rapamycin in complex 1 (mTORC1) (12–14). Another important amino acid transporter, proton-assisted amino acid transporter 1 (PAT1), lies within the lysosomal membrane and is speculated to be important for sensing lysosomal amino acids as well as signaling mTORC1 to the lysosomal membrane to promote the complex's activation (15). These intricate mechanisms illustrate that amino acid transporters are likely critical for facilitating skeletal muscle hypertrophy.

The previously mentioned amino acid transporters share a common transcription factor known as activating transcription factor 4 (ATF4) (16, 17). It could be posited that an increase in ATF4 protein would induce the transcription of the aforementioned amino acid transporters and, thereafter, promote an increase in protein synthesis by increasing intracellular amino acid concentrations. Indeed, several acute human studies have demonstrated that increases in protein synthesis occur in conjunction with an increase in ATF4 protein levels (18–21). However, recent literature suggests that inhibition or knockdown of ATF4 protein promotes protein synthesis, improves muscle strength and quality, and increases muscle fiber diameter in mice (22–24). It has yet to be determined how ATF4 protein is altered following exercise and/or nutrition chronic interventions in human skeletal muscle.

Metabolism of branched-chain amino acids (BCAAs) also plays a role in regulating protein synthesis. Notably, BCAA catabolism begins in the mitochondria with the reversible transamination of BCAAs by the metabolic enzyme branched-chain aminotransferase 2 (BCAT2) which produces branched-chain α-keto acids (BCKAs). BCKAs then undergo irreversible oxidative decarboxylation by the branched-chain α-keto acid dehydrogenase (BCKDH) complex. Importantly, the BCKDH reaction is the rate-limiting enzyme and the commitment step for BCAA metabolism. Regulation of the BCKDH complex is tightly controlled by BCKDH kinase and BCKDH phosphatase. BCKDH kinase phosphorylates the BCKDH complex on the alpha/E1 component (BCKDHα) to inactivate it (25), while BCKDH phosphatase dephosphorylates the BCKDH complex to activate it, resulting in BCAA catabolism. The literature involving the BCKDH complex with regard to humans, exercise, and nutrition is scarce and discrepant. For instance, in rat skeletal muscle it was reported that endurance training decreases skeletal muscle BCKDH kinase protein content (26); however, in human skeletal muscle, endurance training increases BCKDH kinase protein content (27). Interestingly, exercise increases activation of the complex to promote oxidation of BCAAs (28, 29). Furthermore, BCKDH kinase is inactivated by protein or amino acid supplementation promoting increased BCAA oxidation (29). Research on the complex involving exercise has utilized various forms of endurance exercise; however, the effects of resistance exercise and nutrition on regulation of the complex is unknown.

Resistance exercise independently, and with essential amino acid or protein supplementation, has been shown to improve skeletal muscle size, function, and quality in both young and old humans (30–36). Furthermore, acute resistance exercise has been shown to independently increase the mRNA expression and protein content of amino acid transporters (19, 37). Researchers have also demonstrated that amino acid transporter protein content is further improved when a supplement containing amino acids is ingested following exercise (18, 21, 38). Studies in this area, however, have only examined the acute response following resistance exercise, and the chronic response following resistance exercise training has yet to be determined. Therefore, the purpose of this study was to determine how skeletal muscle ATF4 protein levels, amino acid transporters, and metabolic enzymes involved with BCAAs catabolism are altered following resistance exercise training with different nutritional supplementation strategies, and if these changes are related to skeletal muscle fiber hypertrophy. Furthermore, based on these *in vivo* findings, *in vitro* experiments were performed to elucidate potential mechanisms.

## Methods

### Ethical Approval and Participant Screening

Prior to commencing this study, this protocol was reviewed and approved by the Auburn University Institutional Review Board and was in compliance with the Helsinki Declaration (approved protocol #: 15-320 MR 1508). Participants read and signed an informed consent prior to participation. Males that were apparently healthy, free of medical or orthopedic conditions, 19–23 years old, and unaccustomed to resistance training were recruited for participation. Participants stated they had not resistance trained within 6 months, used anabolic steroids, consumed dietary protein in excess of 2.0 g/kg/day regularly, or been consuming dietary supplements.

### Experimental Design

This study is a follow-up analysis from a prior study, and readers are encouraged to read Mobley et al. (39) for complete methods including participant assignment to supplement groups according to whole-body lean tissue mass (assessed by dual-energy x-ray absorptiometry). Importantly, certain groups from that investigation were omitted (i.e., SOY and WPH), given that we felt the included groups (i.e., PLA, LEU, and WPC) would better delineate supplementation-related effects related to leucine and all amino acids (further described in “Supplementation and Dietary Intake”). Briefly, for baseline testing (PRE), participants refrained from physical activity for 96 h, reported to the laboratory hydrated (urine specific gravity <1.020), and 4 h fasted. Participants were then analyzed for body composition via DEXA (General Electric Lunar Prodigy enCORE, version 10.50086; Madison, WI, USA) and donated a skeletal muscle biopsy sample from the vastus lateralis. Two to three days following PRE, initial strength testing was conducted, and these baseline values were used for program prescription. Upon PRE completion, supplementation including placebo (PLA), leucine (LEU), and whey protein concentrate (WPC) was assigned to participants. Participants were instructed to consume supplements twice daily throughout the resistance exercise training protocol, which was completed 3x/week for 12 weeks. Approximately 72 h following each participant's last training session, the participant arrived similar to PRE conditions, underwent DEXA analysis, and donated a second skeletal muscle biopsy sample (POST).

### Skeletal Muscle Biopsy

Skeletal muscle biopsy procedures have been described by our laboratory elsewhere (40, 41). Briefly, participants were instructed to lay supine on a treatment table. Excision of vastus lateralis tissue using a 5 mm Bergstrom Biopsy needle with suction was performed using local anesthesia (1% Xylocaine) and aseptic technique. Tissue was immediately blotted and then distributed for immunohistochemistry (IHC) and molecular analyses. The IHC section was placed in a cryomold with optimal cutting temperature (OCT) media (Electron Microscopy Sciences; Hatfield, PA, USA), slowly frozen in liquid nitrogen-cooled isopentane, and then stored at −80°C until staining procedures. Leftover tissue following IHC partitioning was flash-frozen in liquid nitrogen and stored at −80°C until further analyses. PRE and POST muscle biopsies were taken from the same leg at least 5 cm apart.

### Supplementation and Dietary Intake

Participants were counterbalanced into one of five groups although as previously mentioned only three groups were utilized: placebo (PLA), leucine (LEU), and whey protein concentrate (WPC). Group counterbalancing was based on DEXA lean body mass. Groups differed based on the supplementation provided, and the nutritional breakdown for each supplement is provided in Table 1. Notably, each PLA serving contained 44.4 g of carbohydrate with little leucine and protein, each LEU serving contained 43.1 g of carbohydrate with 2,871 mg of leucine and little protein, and WPC serving contained little carbohydrate with 2,794 mg of leucine and 26.3 g of protein. Each supplement was formulated to contain a similar amount of total energy (kcal) and fat (g) and was blinded to laboratory personnel and participants. Supplements were similar in appearance, taste, texture, and packaging; however, supplement packages only contained a serial number and were double-blinded to the investigators as well as the participants. The serial numbers corresponding to the individual supplements were kept blind to the staff until the conclusion of the study.

**Table 1:** Macronutrient information per serving.

	**PLA**	**LEU**	**WPC**
Energy (kcal)	204	200	184
Total carbohydrate (g)	44.4	43.1	12.0
Dietary fiber (g)	1.6	1.8	1.8
Sugars (g)	6.0	5.1	5.9
Protein (g)	**0.4**	**2.3**	**26.3**
Total fat (g)	2.8	2.0	3.5
Leucine (mg)	**15**	**2,871**	**2,794**

*Bold lettering highlights differences between supplementation groups*.

Participants were instructed to consume two servings per day of their respective supplement. On training days, one serving was consumed immediately following resistance exercise, and the other serving was consumed prior to bed. On non-training days, one serving was consumed between meals, and the other serving was consumed prior to bed. Supplements were mixed in shaker bottles with 500 mL of water, and participants were instructed as to how to blend their supplement. Individual packets were distributed in a 3-week supply, and empty packets were returned to ensure compliance. Participants that did not consume 80% or more of packets were excluded from analyses.

Food logs were filled out by participants at baseline (PRE) and week 12 (POST) of training. Participants were given detailed instructions on how to determine food proportions and thoroughness of meal description needed for accurate assessment. Four daily food records (2 weekdays [Mon–Fri] and 2 weekend days [Sat and Sun]) were submitted by each subject at both PRE and POST. Dietary intake was analyzed using “MyFitnessPal” (MyFitnessPal, Inc., Baltimore, MD, USA). Participants were instructed to not add supplementation to their food logs. Upon completion of the study, supplement nutrition was added to existing totals and then averaged to represent participants' eating habits.

### Resistance Training Program

Strength testing was completed at PRE whereby participants performed a 3-repetition maximum for squat and bench press. Following PRE testing, participants completed 12 weeks of resistance exercise training prescribed 3x/week. Exercises included squat, bench press, deadlift, and bent-over row prescribed at 4 sets of 10 repetitions on Sunday or Monday, 6 sets of 4 repetitions on Tuesday or Wednesday, and 5 sets of 6 repetitions on Thursday or Friday. Relative training intensities were based on PRE testing 3-repetition maximum values and increased weekly with the exception of 2 weeks (weeks 7 and 12) which were dedicated for “deloading” (i.e., a reduction in training volume) to prevent injury and promote recovery. More information regarding progression through the training period can be found within Mobley et al. (39). Participants who missed more than 4 exercise sessions were not included in analyses due to lack of training compliance. POST strength testing was performed following 12 weeks of resistance exercise training whereby participants performed a 3-repetition maximum for squat and bench press.

### Western Blot Analysis

For protein analyses, ~50 mg from each muscle biopsy sample was placed in 1.7 mL microcentrifuge tubes containing 500 μL of ice-cold cell lysis buffer [20 mM Tris-HCl (pH 7.5), 150 mM NaCl, 1 mM Na_2_EDTA, 1 mM EGTA, 1% Triton; Cell Signaling, Danvers, MA, USA] pre-stocked with protease and Tyr/Ser/Thr phosphatase inhibitors (2.5 mM sodium pyrophosphate, 1 mM β-glycerophosphate, 1 mM Na_3_VO_4_, 1 μg/mL leupeptin). Samples were then homogenized by hand via micropestle manipulation, insoluble proteins were removed with centrifugation at 500 *g* for 5 min and obtained sample lysates were batch process-assayed for total protein content using a BCA Protein Assay Kit (Thermo Fisher Scientific). Lysates were then prepared for Western blotting using 4x Laemmli buffer and standardized for protein content at 1.0 μg/μL. Samples were equally loaded onto 10% SDS-polyacrylamide gels (Bio-Rad, Hercules, CA, USA) and subjected to electrophoresis (150 V for 60 min) using pre-made 1x SDS-PAGE running buffer (Ameresco). Proteins were then transferred (200 mA for 2 h) to polyvinylidene difluoride membranes (Bio-Rad), Ponceau S stained, and imaged to ensure equal protein loading between lanes. Membranes were then blocked for 1 h at room temperature with 5% non-fat milk powder in Tris-buffered saline with 0.1% Tween-20 (TBST; Ameresco, Solon, OH, USA). Following blocking, membranes were incubated in primary antibodies including: ATF4 (1:2,000; Abcam, ab1371, Cambridge, MA, USA), LAT1 (1:1,000; Cell Signaling, #5347, Danvers, MA, USA), PAT1 (1:200; Santa Cruz Biotechnology, sc-368553, Dallas, TX, USA), SNAT2 (1:1,000; Santa Cruz Biotechnology, sc-514037), BCAT2 (1:1,000; Abcam, ab95967), BCKDHα (1:1,000; Genetex, GTX45109, Irvine, CA, USA), eGFP (1:1,000; Bioss Inc.; bs-2194R, Boston, MA, USA), and Puromycin (1:1,000; MilliporeSigma; MABE342; Burlington, MA, USA). Primary antibody was incubated with membranes overnight at 4°C in TBST with 5% bovine serum albumin (BSA). The following day, membranes were incubated with horseradish peroxidase-conjugated anti-rabbit or anti-mouse IgG (1:2,000, Cell Signaling) in TBST with 5% BSA at room temperature for 1 h. Membrane development was performed using an enhanced chemiluminescent reagent (Luminata Forte HRP substrate; MilliporeSigma). Band densitometry was performed using a gel documentation system and associated densitometry software (UVP, Upland, CA, USA). Densitometry values for all protein targets were normalized to Ponceau S densities. Each participant's PRE and POST sample was run in continuous lanes and on the same gel. All values for a given protein target were normalized to each participant's PRE value to provide fold change scores.

Notably, membranes for various targets were cut into upper and lower sections for antibody incubations; for example, one set of membranes were cut in half ~55 kDa and the top half membrane was incubated with ATF4 primary antibody solution (target band ~63 kDa), whereas the bottom half membrane was incubated with LAT1 primary antibody solution (target band ~48 kDa). Additionally, bottom half membranes were re-probed for BCKDHα by exposing them to commercial stripping buffer following LAT1 quantification (ThermoFisher Scientific; 21059), and we ensured the stripping buffer removed all visible LAT1 bands prior to reprobing for BCKDHα. In these situations, the same Ponceau images were used to normalize ATF4, LAT1, and BCKDHα data (this can be seen in **Figures 2A,B,F** *where the same subjects were used for representative images*).

### Immunohistochemical Analysis

Immunohistochemistry (IHC) was performed to determine muscle fiber cross-sectional area (fCSA) and LAT1 protein content, and these procedures have been previously described (42). Briefly, skeletal muscle biopsy samples frozen in OCT media were sectioned to 10 μm using a cryotome (Leica, Biosystems; Buffalo Grove, IL, USA). All samples were sectioned and stored at −80°C until staining occurred. To determine fCSA, sections were air-dried at room temperature for 30 min, fixed with 10% formalin for 10 min, permeabilized in phosphate-buffered saline (PBS) that contained 0.5% Triton X-100, and blocked with 100% Pierce Super Blocker (ThermoFisher Scientific; #37515) for 25 min. Sections were then washed with PBS and incubated for 1 h in primary antibody solution containing rabbit anti-dystrophin (ThermoFisher Scientific; PA5-32388) at 1:100 dilution and mouse anti-myosin II (Hybridoma Bank; SC71) at 1:100 dilution in blocking solution. Sections were then washed for 5 min in PBS and incubated with secondary antibody solution containing Texas Red anti-rabbit (Vector Laboratories; Burlingame, CA, USA) at 1:100 dilution and Alexa Flour 488 anti-mouse (ThermoFisher Scientific) at 1:100 dilution in blocking solution. Notably, it was possible to capture fiber-type distribution using this technique. As a result, the “mean fCSA” metric used as a dependent measure herein is the change in fiber cross sectional area relative to that individual's fiber type distribution. Alternatively stated, the change in Type I fibers was made relative to the participant's respective fiber type distribution and was then added to the change in Type II fibers relative to the respective fiber type distribution for a “mean fCSA.”

To determine LAT1 protein content, methods from Hodson et al. (43) were utilized with the exception of the fixation step as this step was found to confound the LAT1 signal during pilot staining. This alteration may explain the difference in staining intensity between their investigation and the present investigation. Briefly, sections were air-dried for 10 min, washed 3 times in PBS for 5 min each, and then blocked using Superblocker (ThermoFisher Scientific; #37515) for 30 min. Sections were then incubated with primary antibody solution containing mouse anti-dystrophin (ThermoFisher Scientific; PA5-32388) at 1:100 dilution and rabbit anti-LAT1 (Abcam, #85226) at 1:100 dilution (43). Notably, these experiments were duplicated with the same LAT1 primary antibody used during western blotting (Cell Signaling, #5347); however, this antibody was not specifically designated for IHC per manufacturer recommendations. As a result, only data yielded from the working antibody (Abcam, #85226) are presented herein. Sections were incubated in primary antibody solution for 2 h, washed in PBS, and incubated for 1 h with secondary antibody containing Texas Red anti-rabbit (Vector Laboratories; TI-1000) and Alexa Fluor 488 anti-mouse (Thermo Fisher Scientific; A-11001). Sections were then washed with PBS, mounted, and stored in the dark at 4°C until fluorescent images were taken. Notably, preliminary experimentation determined the antibodies used did not display cross-reactivity.

Digital 20x and 40x images were obtained using a fluorescence microscope (Nikon Instruments; Melville, NY, USA). Four sections (3 experimental and 1 negative control) per participant were mounted on each slide. Each section was separated by ~100 μm. Two images were taken per experimental section for a total of 6 images per participant per timepoint, and at least 50 fibers were quantified per image. The counts from the 6 images were averaged and used during statistical analysis. Approximate exposure times were 600 ms for red and green imaging. This staining method allowed the identification of cell membranes (detected by the FITC filter; green) and LAT1 protein (detected by the TRITC filter; red). Measurements for type I fCSA, type II fCSA, and mean fCSA and LAT1 protein were conducted using the open-sourced software CellProfiler^TM^ (44) whereby the number of pixels counted within the border of each muscle fiber were converted to total area. Threshold settings for quantifying LAT1 protein were first validated by manually counting the number of LAT1 protein particles, and then adjusting the threshold settings to meet the manually counted LAT1 protein particles.

### Cell Culture

C2C12 myoblasts, passage number 5–8, were maintained in Dulbecco's Modified Eagle Medium (DMEM; Gibco; #11965092) supplemented with 10% Fetal Bovine Serum (FBS), 1% penicillin-streptomycin, and 0.1% gentamycin. Myoblasts were used instead of myotubes given the well-known difficulty in transfecting myotubes without a virus. Cells were seeded in 6-well plates (Corning; #3335) at 0.3 x 10^6^ in 3 mL of growth medium per well. Once myoblasts reached 60–70% confluence, myoblasts were transfected with a LAT1 plasmid (Genecopoeia, Rockville, MD, USA; EX-Mm05301-M98) via Lipofectamine 2000 (Invitrogen; #11668019) using manufacturer's protocols. Control cells were transfected with an empty vector (Genecopoeia, EX-NEG-M98) using identical methods. Both plasmids utilized a CMV promoter within the pReceiver-M98 vector and contained an eGFP reporter. Twenty-four hours later cells were pulse-labeled with 1 μM puromycin hydrochloride (Ameresco) in PBS to assess relative protein synthesis levels. Puromycin was incubated with cells for 30 min, medium was removed, cells were washed with ice-cold PBS, and harvested in ice-cold cell lysis buffer that was previously described. Western blotting methods for cell culture samples was identical to methods previously described. Proteasome activity assays on cell lysates were completed using a commercially available fluorometric kit according to manufacturer's instructions (MilliporeSigma; APT280).

### Liquid Chromatography-Mass Spectrometry

Intracellular amino acid concentrations from C2C12 myoblasts were determined by using a liquid chromatography–electrospray ionization–mass spectrometry system (QTrap 5500 MS; AB Sciex; Framingham, MA, USA) with liquid chromatography device ExpressHT Ultra LC (Eksigent Div; AB Sciex). Samples were standardized with 0.1 N HCl that contained a stable isotopomer of every amino acid as an internal standard. Samples were transfer pipetted onto strong-cation-exchange drip columns. Columns were washed with water and eluted with 2.5 mol/L ammonia. Eluates were desolvated in a centrifugal evaporator. Solid-residue tubes were capped for storage in the dark at room temperature. Within 3 d of liquid chromatography–electrospray ionization–mass spectrometry analysis, samples were derivatized with 9-fluorenylmethoxycarbonyl (FMOC) and subsequently neutralized, after which sample solutions were injected onto a 0.5 × 100 mm HALO C18 column (Eksigent Div; AB Sciex) and kept at 35°C. Analytes were eluted with a segmentally linear gradient from 35 to 85% acetonitrile in water supplemented with ammonium acetate to 10 mmol/L and 5% isopropanol. Detection was performed by using electrospray triplequadrupole–tandem mass spectrometry in multiple-reaction monitoring mode. FMOC amino acid derivatives were fragmented in the collision cell for detection of either free aminoacyl anions or a fragment larger by 26 atom mass units (coming from the FMOC derivative), whichever gave the highest sensitivity. Thus, monitoring occurred for each amino acid, and internal standards. We used the SignalFinder algorithm in MultiQuant software (version 2.1; ABScieix) to quantify peaks.

### Statistical Analyses

Certain data (e.g., participant characteristics, muscle fCSA, nutrition information) have been previously analyzed with additional supplementation groups, different sample sizes, and different statistical methods (39); however, these data have been reanalyzed herein using the three groups of interest, sample sizes related to this investigation, and alternate statistical methods which are described below.

Various participant characteristics were compared between groups using one-way ANOVAs. All dependent variables related to IHC, Western blotting, certain participant characteristics, and nutritional data were assessed using two-way repeated measures ANOVAs to test differences between groups (PLA, WPC, and LEU) and over time (PRE and POST). Fold-change was utilized for protein expression and IHC whereby POST was divided by PRE within each participant. This method was chosen based on past literature (18–20). Fold changes from all participants in a given group were then averaged. Given PRE was normalized to 1 with no standard deviation, the main effect for group *p*-value and group by time interaction *p*-value were identical; therefore, only the main effect for group and the main effect for time *p*-values were reported. Unlike this investigation, the aforementioned studies utilized 3 or more time points which allowed for numerical differences between the group main effect and group by time interaction *p*-values. Again, this method was chosen *a priori* based on the previous literature. Pearson correlations were also performed between all participant's individual change in fCSA and their fold change in western blot proteins to determine if relationships existed between these variables and, if trending toward significance or significant, was followed up with a simple linear regression analysis for each group to discern potential group differences. Cell culture data were analyzed using independent samples *t*-tests between conditions (LAT1 overexpression or control). Cell culture data fold change values were generated by dividing each sample value by the CTL average.

Normality was tested for all dependent variables using a Shapiro-Wilk test. Outliers were removed if they were ±3 times the interquartile range. When appropriate, Levene's test for equality of variances was determined. Assumptions testing was seldom violated; however, data were not manipulated due to the robustness of the ANOVA test, and the majority of the groups within the analysis did not violate this assumption. If a significant f-value was found following an ANOVA test, further statistical analysis was conducted using a LSD *post-hoc* comparison given the exploratory nature of this investigation. For regression analysis, heteroscedasticity, and residuals were analyzed. Sample sizes are noted in each figure legend and vary due to tissue and sample limitations. All data are presented as mean ± standard deviation values, trending statistical significance was established as 0.050 < *p* < 0.100, and statistical significance was established as *p* < 0.050. All statistical analyses were performed using SPSS version 24.0 (Chicago, IL, USA).

## Results

### Participant Characteristics and Nutrition Information

Participant characteristics are reported in Table 2. As noted in the parent publication by Mobley et al. (39), 18 participants were enrolled in the PLA group, and 15 participants finished the study due to supplement and workout compliance. The LEU group had 17 initial enrollees, and 15 participants finished the study due to supplement and workout compliance. The WPC group had 19 initial enrollees, and 17 participants finished the study due to supplement and workout compliance. Due to tissue limitations, however, samples sizes are reported in each figure legend.

**Table 2:** Participant characteristics prior to and following resistance training.

	**PLA (*****n*** **=** **12)**		**LEU (*****n*** **=** **13–14)**		**WPC (*****n*** **=** **15–17)**		**ANOVA** ***p*****-value**		
	**PRE**	**POST**	**PRE**	**POST**	**PRE**	**POST**	**Group**	**Time**	**G × T**
Age (years)	21 ± 1		20 ± 1		21 ± 2		0.336		
Height (cm)	182.2 ± 9.1		179.4 ± 4.8		179.0 ± 6.4		0.444		
Total volume lifted (kg)	3,07,020 ± 34,937		3,24,637 ± 60,633		3,30,086 ± 51,791		0.414		
Fat mass (kg)	17.4 ± 4.9	17.8 ± 4.7	15.3 ± 5.8	15.8 ± 6.4	19.5 ± 8.6	18.7 ± 8.3	0.366	0.930	0.120
Fat free mass (kg)	57.3 ± 6.5	60.2 ± 6.4	56.8 ± 5.1	59.3 ± 4.9	59.0 ± 7.0	61.3 ± 6.9	0.625	**<0.001**	0.664
Total mass (kg)	74.8 ± 10.0	78.0 ± 9.5	72.1 ± 9.5	75.1 ± 10.3	78.5 ± 13.0	80.0 ± 13.4	0.387	**<0.001**	0.212
VL thickness (cm)	2.28 ± 0.30	2.74 ± 0.32	2.41 ± 0.24	2.77 ± 0.30	2.49 ± 0.23	2.96 ± 0.29	0.080	**<0.001**	0.446
3RM–Squat (kg)	67 ± 12^a^	108 ± 15^*^	85 ± 20^b^	112 ± 22^*^	83 ± 21^b^	118 ± 23^*^	0.177	**<0.001**	**0.031**

* <0.050 compared to PRE within group only if the G × T interaction was significant.

*Differing letters represent significant differences between groups within each time point. Bold lettering denotes a significant p-value*.

Notably, differences did not exist between groups for age, height, or total volume lifted (*p* > 0.050). Moreover, fat mass, fat free mass, total mass, or vastus lateralis (VL) thickness were not different between groups at the beginning of the study (*p* > 0.050). Fat free mass, total mass, and VL thickness increased following resistance training (*p* < 0.001). Performance measured by squat demonstrated a group by time interaction (*p* = 0.031) whereby PLA squatted less than LEU and WPC at PRE; however, no differences existed at POST. All groups increased squat performance from PRE to POST (*p* < 0.001).

Self-reported nutrient intake data are reported in Table 3. All groups increased nutrient intake shown by a significant time effect for consumption of absolute and relative carbohydrates, protein, fat, and total energy following training (*p* < 0.005). WPC consumed less absolute and relative carbohydrates (*p* < 0.050) and more absolute and relative protein (*p* < 0.050) compared to PLA and LEU; albeit, all groups consumed statistically similar amounts of absolute and relative total energy.

**Table 3:** Nutrition information prior to and following resistance training.

	**PLA (*****n*** **=** **12)**		**LEU (*****n*** **=** **14)**		**WPC (*****n*** **=** **17)**		**ANOVA** ***p*****-value**		
	**PRE**	**POST**	**PRE**	**POST**	**PRE**	**POST**	**Group**	**Time**	**G × T**
Carbohydrates (g)	225 ± 92	348 ± 123^*^^a^	206 ± 62	310 ± 79^*^^a^	215 ± 59	244 ± 64^b^	0.100	**<0.001**	**0.009**
Carbohydrates (g/kg)	2.9 ± 1.1	4.3 ± 1.3^*^^a^	2.8 ± 1.1	4.1 ± 1.4^*^^a^	2.8 ± 1.0	3.0 ± 1.0^b^	0.143	**<0.001**	**0.010**
Protein (g)	80 ± 20	112 ± 49^*^^a^	87 ± 24	108 ± 36^*^^a^	88 ± 26	145 ± 24^*^^b^	0.094	**<0.001**	**0.009**
Protein (g/kg)	1.1 ± 0.2	1.4 ± 0.5^a^	1.2 ± 0.3	1.4 ± 0.5^a^	1.1 ± 0.3	1.8 ± 0.4^b^	0.294	**<0.001**	**0.009**
Fat (g)	78 ± 21	111 ± 52	73 ± 18	92 ± 24	71 ± 22	93 ± 51	0.354	**0.002**	0.744
Fat (g/kg)	1.0 ± 0.3	1.4 ± 0.5	1.0 ± 0.3	1.2 ± 0.4	0.9 ± 0.4	1.2 ± 0.7	0.480	**0.005**	0.851
Total energy (kcal)	1,969 ± 631	2,830 ± 1,006	1,835 ± 435	2,488 ± 495	1,866 ± 475	2,389 ± 729	0.347	**<0.001**	0.519
Total energy (kcal/kg)	25.4 ± 7.7	34.8 ± 10.1	24.9 ± 7.3	32.6 ± 8.7	23.7 ± 8.1	29.6 ± 11.0	0.454	**<0.001**	0.631

* < 0.050 compared to PRE within group only if the G × T interaction was significant.

*Differing letters represent significant differences between groups within each time point. Bold lettering denotes a significant p-value*.

### Fiber Cross Sectional Area

**Figure 1:**
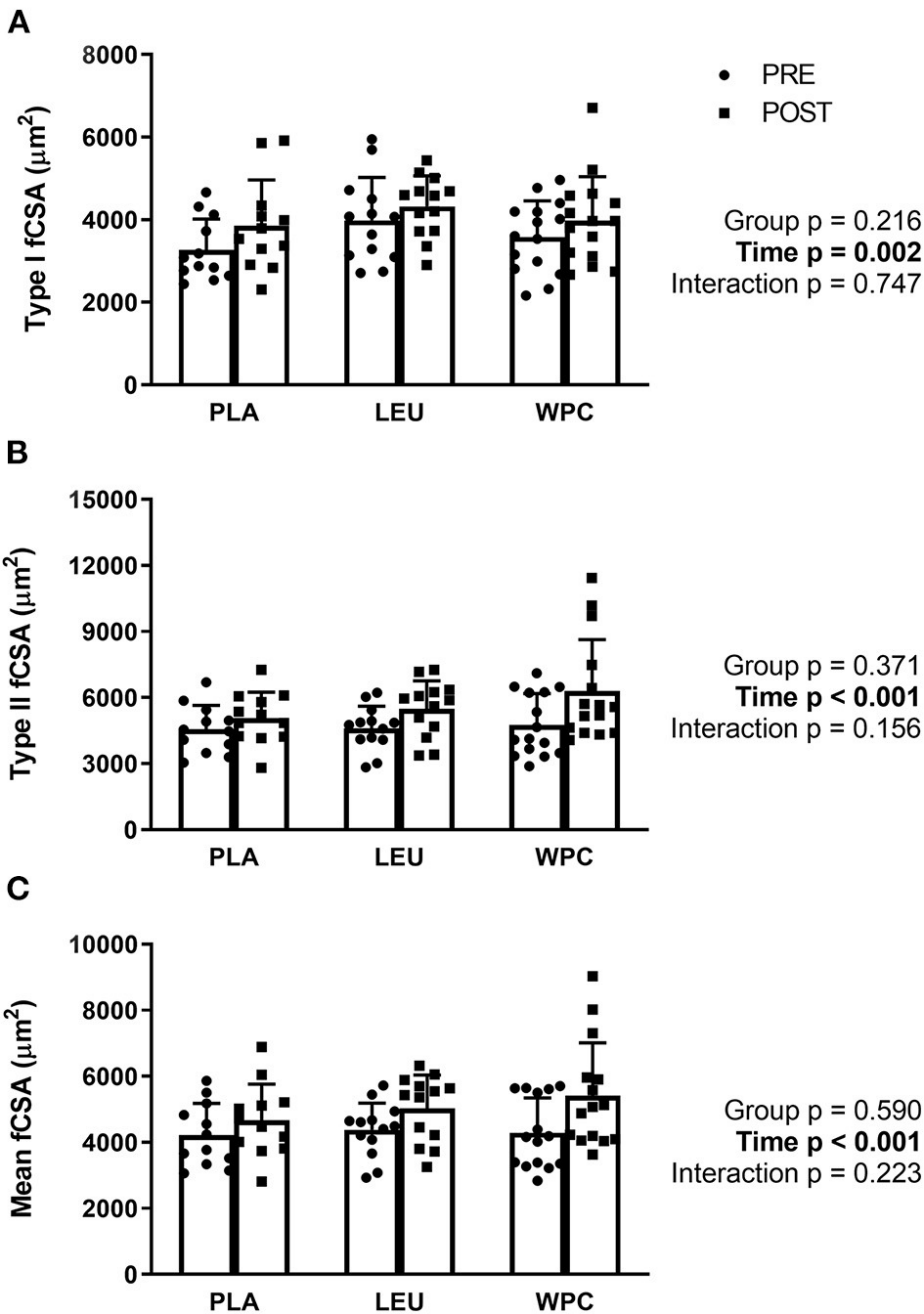
Muscle fiber cross-sectional area (fCSA) increases with resistance exercise training independent of supplementation group. Skeletal muscle type I **(A)**, type II **(B)**, and mean **(C)** fCSA increased following 12 weeks of total-body resistance exercise training independent of consuming placebo (PLA), leucine (LEU), or whey protein concentrate (WPC). Open columns designate pre-training values (PRE), and closed columns designate post-training values (POST). Data are represented as mean ± standard deviation. Sample size for each group is PLA = 12, LEU = 13, and WPC = 15.

Following 12 weeks of total-body resistance exercise training, type I fibers increased in fiber cross-sectional area (fCSA) from 3,618 ± 919 μm^2^ to 4,052 ± 982 μm^2^ which was demonstrated by a significant time effect (*p* = 0.002; Figure 1A). Likewise, Type II fibers increased in fCSA following training from 4,648 ± 1,170 μm^2^ to 5,661 ± 1,768 μm^2^ shown by a significant time effect (*p* < 0.001; Figure 1B). Lastly, growth of Type I and II fibers relative to each participant's fiber type composition (mean fCSA) increased following training from 4,297 ± 932 μm^2^ to 5,063 ± 1,292 μm^2^ demonstrated by a significant time effect (*p* < 0.001; Figure 1C). Neither type I, type II, or mean fCSA revealed a group effect (*p* > 0.050) or group x time interaction (*p* > 0.050).

### Protein Abundance

**Figure 2:**
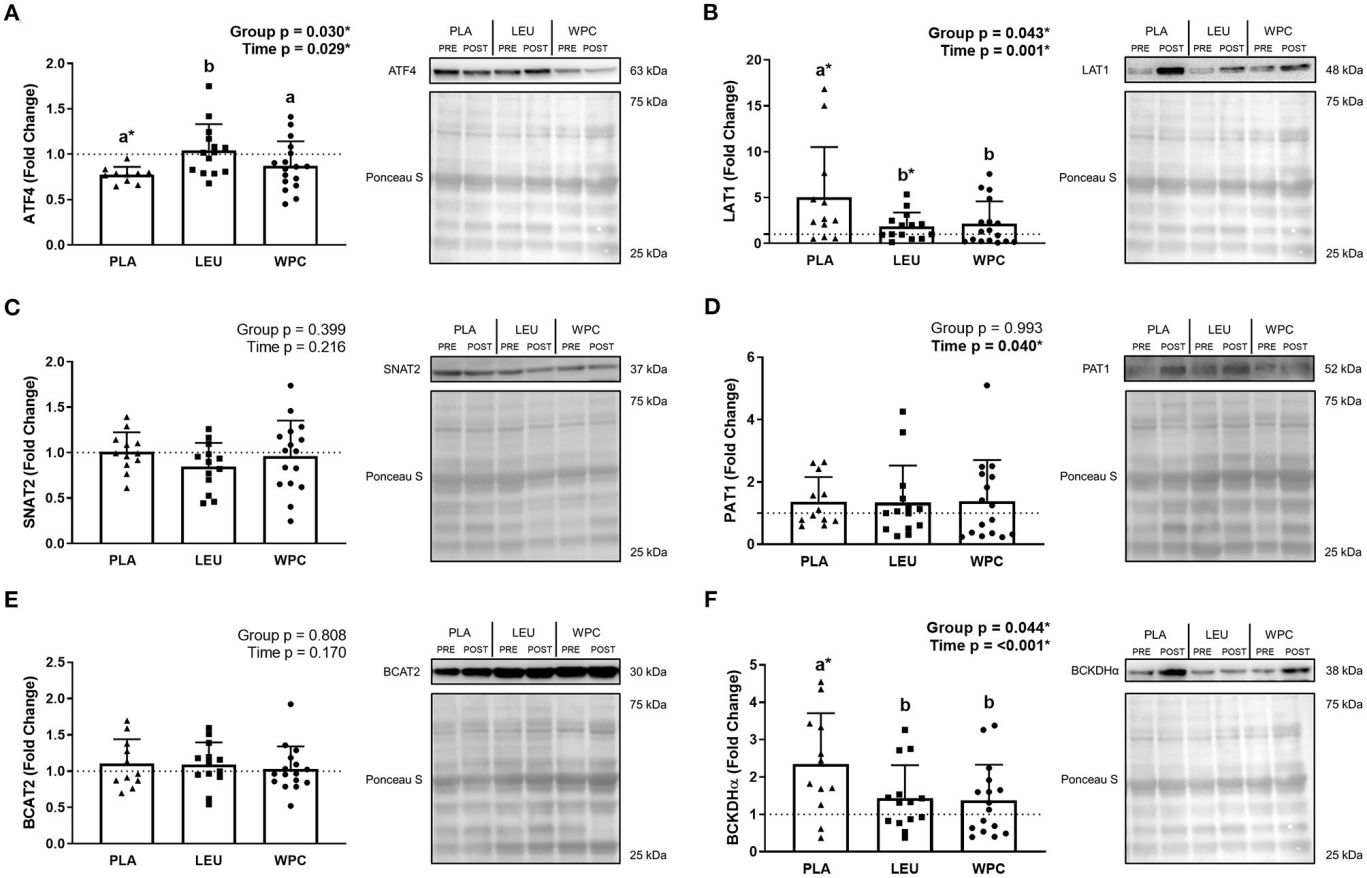
The protein content of amino acid transporters and metabolic enzymes are altered following 12 weeks of resistance exercise training. The fold change from PRE, designated as the dashed line at 1.00, for ATF4 **(A)**, LAT1 **(B)**, SNAT2 **(C)**, PAT1 **(D)**, BCAT2 **(E)**, and BCKDHα **(F)** protein content for each group. A representative image for each protein and loading control (i.e., Ponceau S stain) accompanies each panel. Differing letters represent significant differences between groups within each time point. *p < 0.050 compared to PRE within group only if the G × T interaction was significant. Data are represented as mean ± standard deviation. Sample size for each group is PLA = 10–12, LEU = 13–14, and WPC = 16–17.

ATF4 protein (Figure 2A) demonstrated a group effect (*p* = 0.030) whereby the fold change in PLA (0.77 ± 0.09) was less than LEU (1.04 ± 0.29; *p* = 0.011), but not different than WPC (0.87 ± 0.27; *p* = 0.308), although WPC trended to be lower than LEU (*p* = 0.061). ATF4 protein decreased following training which was demonstrated by a significant time effect (*p* = 0.029; 1.00 to 0.91); albeit, only PLA significantly decreased from PRE to POST (1.00 to 0.77 ± 0.09) while LEU and WPC were unaltered (*p* > 0.050).

LAT1 protein (Figure 2B) revealed a group effect (*p* = 0.043) whereby the fold change in PLA (5.01 ± 5.50) was higher than LEU (1.87 ± 1.49; *p* = 0.023) and WPC (2.15 ± 2.43; *p* = 0.031), but LEU and WPC were not significantly different from one another. LAT1 protein also increased following training which was demonstrated by a significant time effect (*p* = 0.001; 1.00 to 2.86). PLA (1.00 to 5.01 ± 5.50) and LEU (1.00 to 1.87 ± 1.49) significantly increased from PRE to POST while WPC was unaltered (*p* > 0.050).

SNAT2 protein (Figure 2C) was unaltered following training or by supplementation shown by a non-significant group effect (*p* = 0.399) and time effect (*p* = 0.216). Similarly, BCAT2 protein (Figure 2E) was unaltered by training (time *p* = 0.170) or supplementation (group *p* = 0.808). PAT1 protein (Figure 2D) increased with training shown by a significant time effect (*p* = 0.040; 1.00 to 1.37) but not with supplementation shown by a non-significant group effect (*p* = 0.993).

BCKDHα protein (Figure 2F) demonstrated a group effect (*p* = 0.044) whereby the fold change in PLA (2.35 ± 1.36) was greater than LEU (1.44 ± 0.88; *p* = 0.035) and WPC (1.38 ± 0.95; *p* = 0.022), but LEU and WPC were not significantly different from each other. BCKDHα protein also increased following training which was demonstrated by a significant time effect (*p* < 0.001; 1.00 to 1.78), although only PLA significantly increased from PRE to POST (1.00 to 2.35 ± 1.36) while LEU and WPC were unaltered (*p* > 0.050).

### Immunohistochemistry

**Figure 3:**
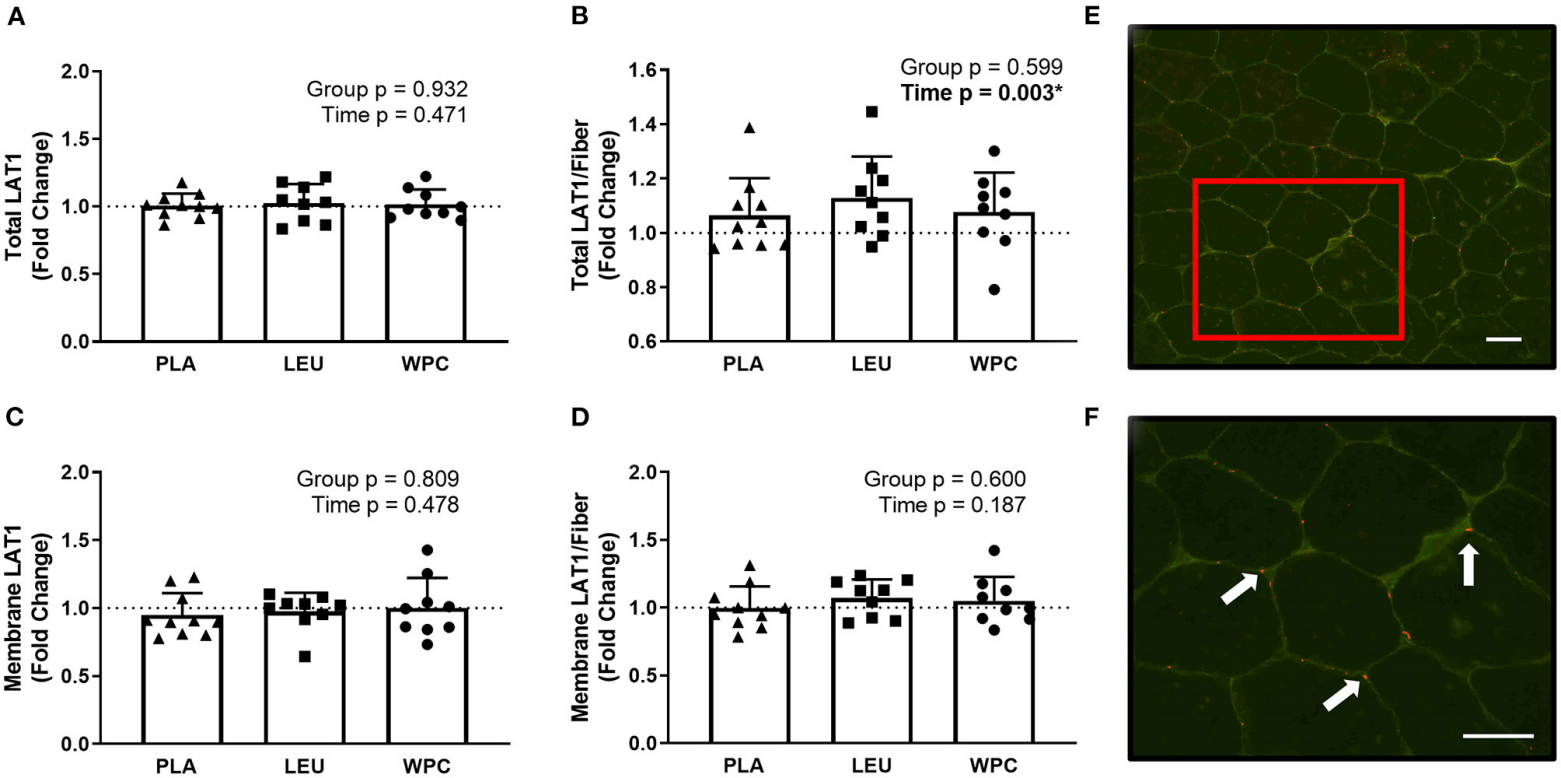
LAT1 protein increases per skeletal muscle fiber with resistance exercise training but the increase in protein is not located on the membrane. The fold change from PRE, designated as the dashed line at 1.00, for Total LAT1 **(A)**, Total LAT1 per fiber **(B)**, Membrane LAT1 **(C)**, and Membrane LAT1 per fiber **(D)** protein content for each group using immunohistochemistry. A representative image showing green-stained dystrophin and red-stained LAT1 protein using 20x magnification **(E)**; the inset in E is magnified using 40x magnification **(F)** and the arrows point toward LAT1 protein. The scale bar in both images is 100 μm. *A significant effect. Data are represented as mean ± standard deviation. Sample size for each group is PLA = 10, LEU = 9, and WPC = 9.

To confirm our LAT1 western blot data, IHC for LAT1 was performed. Two separate antibodies for LAT1 (Abcam, #85226; Cell Signaling, #5347) were used and analyzed identically but did not produce congruent results. In fact, no correlation existed for the same metric between the two different antibodies (*p* > 0.050) (*data not shown)*. In this regard, one antibody (Abcam, #85226) was designated for immunohistochemistry per manufacturer's recommendations and is presented herein (Figure 3), while the other antibody was not designated for immunohistochemistry (Cell Signaling, #5347). Results from this antibody (Cell Signaling, #5347) are shown in Supplementary Figure 1. Using the same dependent metrics for immunohistochemistry listed below, there were no significant findings using this antibody (Cell Signaling, #5347) (*p* > 0.050).

Using the appropriate antibody (Abcam, #85226), staining for total LAT1 protein (sarcoplasmic and membrane LAT1; Figure 3A), membrane LAT1 protein (Figure 3C), or membrane LAT1/fiber (Figure 3D) did not reveal a group effect (*p* > 0.050) or time effect (*p* > 0.050). Total LAT1/fiber did not demonstrate a group effect (*p* = 0.599) but did show a time effect whereby Total LAT1/fiber increased from PRE to POST (*p* = 0.003; 1.00 to 1.09 ± 0.14; Figure 3B).

### Correlations and Regression

To determine if the change in amino acid transporters or metabolic enzymes was indicative of hypertrophy a correlation table was created and is shown in Table 4. Notably, changes in measured proteins were not indicative of the change in Type I fCSA, Type II fCSA, or mean fCSA; however, the change in ATF4 protein trended toward a significant (0.050 < *p* < 0.010) weak correlation (*r* = 0.287–0.314) for changes in measures of fCSA. Moreover, PRE raw-value protein content for each measured protein was not correlated with the change in Type I fCSA, Type II fCSA, or mean fCSA (*p* > 0.100) (*data not shown*).

**Table 4:** Correlations for the change in fCSA and the fold change in proteins.

	**Δ** **Type I fCSA**		**Δ** **Type II fCSA**		**Δ** **Mean fCSA**	
	***r*-value**	***p*-value**	***r*-value**	***p*-value**	***r*-value**	***p*-value**
Δ LAT1	−0.079	0.627	−0.163	0.313	−0.165	0.309
Δ SNAT2	0.023	0.891	0.167	0.316	0.104	0.533
Δ PAT1	−0.066	0.690	−0.068	0.681	−0.065	0.692
Δ ATF4	0.287	0.081	0.305	0.063	0.314	0.055
Δ BCAT2	0.164	0.331	0.199	0.238	0.160	0.343
Δ BCKDHA	0.021	0.899	−0.043	0.796	−0.071	0.667

*Sample size for each correlation was 37–40*.

**Figure 4:**
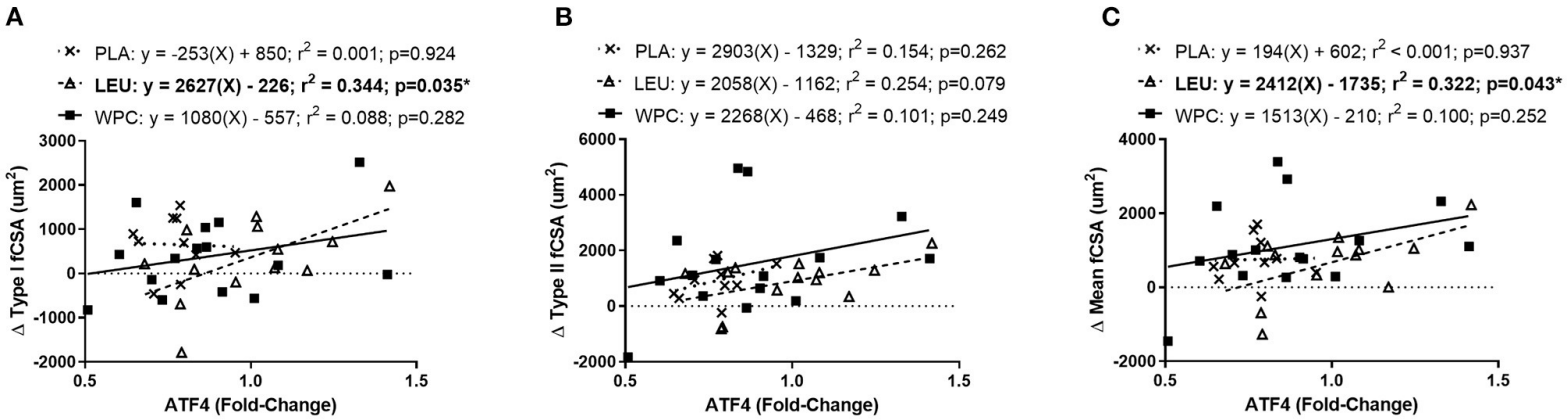
The change in ATF4 protein predicts the change in fCSA only in LEU following 12 weeks of resistance exercise training. Regression analysis was completed using the change in ATF4 protein to predict the change in Type I **(A)**, Type II **(B)**, and mean **(C)** fCSA. Each panel contains each group's predictive equation, coefficient of determination (*r*^2^), and *p*-value. Best fit lines are displayed in each graph. *A significant prediction. Sample size for each group is PLA = 12, LEU = 13, and WPC = 15.

To delineate if the trending correlation between the change in ATF4 protein content and the change in fCSA was skewed by a certain group, a simple linear regression analysis was performed for each group. Indeed, when the change in ATF4 protein was used to predict the change Type I fCSA, a significant (*p* = 0.035) coefficient of determination (*r*^2^ = 0.344) was found for LEU but not for PLA or WPC (Figure 4A). The change in Type II fCSA was not significantly predicted when using the change in ATF4 protein as the predictor (*p* = 0.079; Figure 4B). When the change in ATF4 protein was used to predict the change in mean fCSA, LEU demonstrated a significant (*p* = 0.043) coefficient of determination (*r*^2^ = 0.322; Figure 4C).

### Cell Culture

**Figure 5:**
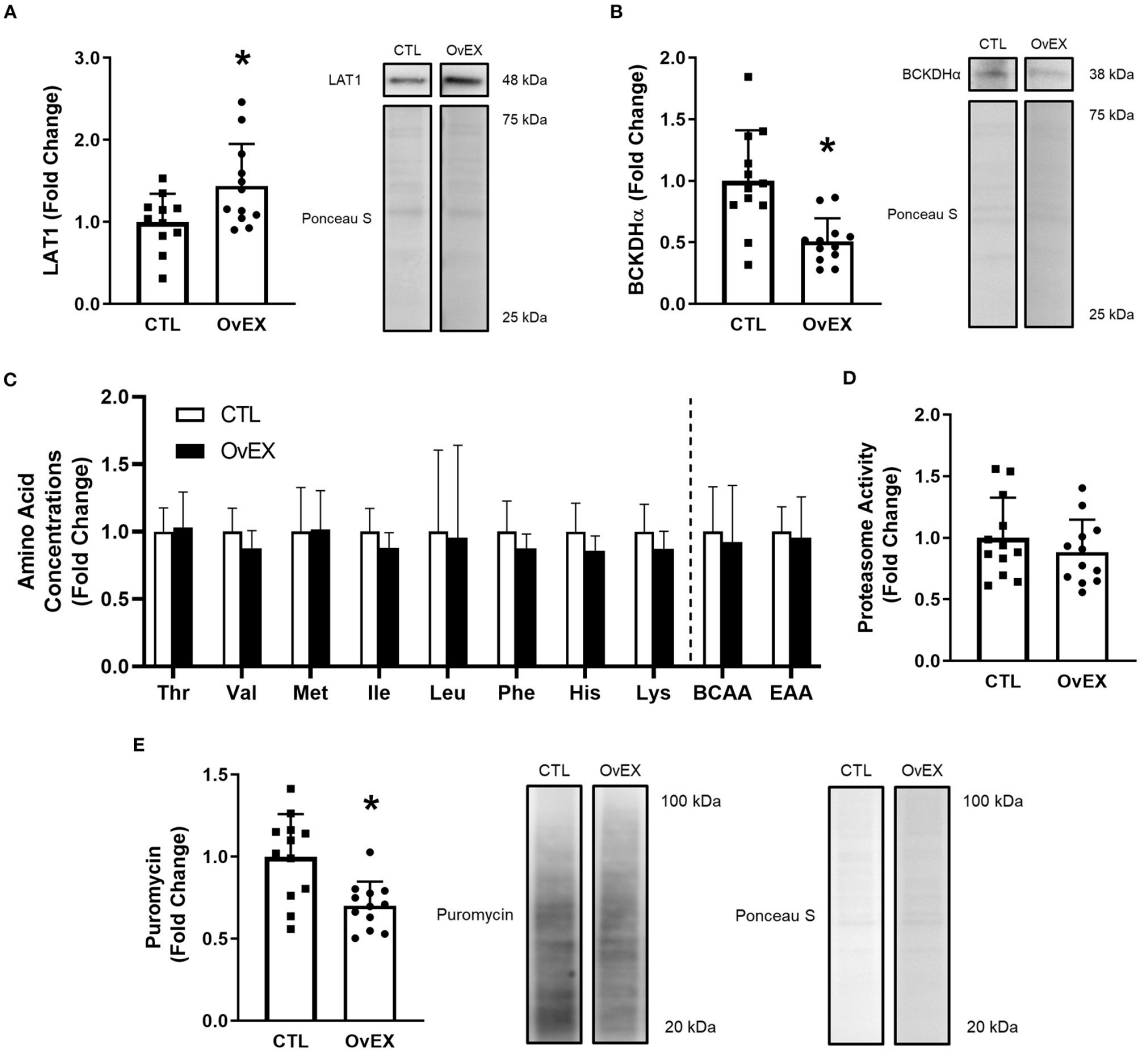
LAT1 protein overexpression in C2C12 myoblasts decreases protein synthesis. Overexpression of LAT1 protein (OvEX) increases LAT1 protein **(A)** but decreases BCKDHα protein **(B)** compared to control (CTL). Amino acid concentrations from whole cell lysates **(C)** as well as proteasome activity **(D)** were unaltered by LAT1 protein overexpression. Puromycin incorporation, a surrogate for protein synthesis, decreased following LAT1 overexpression **(E)**. Representative images for each measured protein accompany each panel. In panel C open columns represent CTL and closed columns represent OvEX. *p < 0.050 compared to control. Sample sizes for each treatment group was CTL = 11–12 and OvEX = 12 with the exception of **(C)** as each group's sample size was 8.

LAT1 protein was overexpressed (OvEX) in C2C12 myoblasts compared to controls (CTL) (*p* = 0.026; Figure 5A). Given the plasmid construct contained eGFP, this protein was also quantified using western blotting and was not different between CTL and OvEX (*p* = 0.977) suggesting transfection efficiency was equal between groups (*data not shown)*. BCKDHα protein decreased with LAT1 protein overexpression compared to CTL (*p* = 0.001; Figure 5B). Using LCMS to determine amino acid concentrations within cell lysates, LAT1 protein overexpression did not alter the concentration of Thr, Val, Met, Ile, Leu, Phe, His, Lys, total BCAAs, or total EAAs (Figure 5C). Protein synthesis measured by puromycin incorporation was lower when LAT1 protein was overexpressed (*p* = 0.002; Figure 5E); however, proteasome activity was not altered (*p* = 0.347; Figure 5D).

## Discussion

Resistance exercise training is a potent anabolic stimulus for promoting skeletal muscle growth and when paired with protein or amino acid supplementation, skeletal muscle growth is optimized (39, 45). Both resistance exercise and protein supplementation independently, and in concert, alter skeletal muscle amino acid transporters and enzymes involved with amino acid metabolism (29, 38). The current investigation demonstrates that amino acid transporters and enzymes involved with BCAA metabolism are altered following 12 weeks of total-body resistance exercise training. Our *in vitro* findings demonstrate that perturbations in LAT1 protein levels can lead to appreciable alterations in protein synthesis and amino acid metabolism.

Each group utilized in the present study consumed a unique supplement and, as expected, PLA and LEU ingested more carbohydrates, but less protein, than WPC. Importantly, absolute and relative total energy consumption was not different between groups given increased energy consumption provides more energy to meet metabolic demand or energy-intensive processes such as protein synthesis. Furthermore, the total amount of weight lifted (i.e., training volume) was not different between groups. Even though supplementation was different between groups, all groups equally increased muscle strength and hypertrophy which was measured by 3-RM squat and ultrasound, respectively. Furthermore, all groups equally increased type I, type II, and mean fCSA with training. Taken together, these results suggest the differences in molecular markers are specific to the supplementation provided.

Several studies have demonstrated skeletal muscle LAT1 protein levels acutely increase following amino acid ingestion, resistance exercise, or both together (18, 19, 38). LAT1 protein is also influenced by bed rest as 7 d of bed rest attenuated the increase in LAT1 protein compared to control (20). The acute increase in amino acid transporters in response to resistance exercise appears transient given that LAT1 protein is not different 72 h following 3 bouts of endurance or resistance exercise compared to basal levels (37) suggesting the findings herein are a resistance exercise training effect. Following 12 weeks of resistance exercise training, LAT1 protein increased and, unexpectedly, increased the most in PLA. This unexpected result may be a result of the significant downregulation of ATF4 protein in the PLA group. These findings regarding the ATF4 protein data expand and corroborate prior literature. First, ATF4 expression is upregulated during periods of amino acid or energy deficiency such as exercise (24). In this regard, Drummond et al. found an increase in ATF4 protein following resistance exercise (19); however, an unexpected and similar increase in ATF4 protein has also been observed following amino acid ingestion (18). Second, ATF4 has been implicated in promoting muscle atrophy (22, 24) and, therefore, a reduction in ATF4 protein content would promote hypertrophy, which is corroborated herein by the significant reduction in ATF4 protein seen in PLA. It is plausible to suspect an initial increase in ATF4 protein during the first weeks of training promoted an increase in LAT1 protein (as well as PAT1 protein); however, as training progressed, ATF4 protein content may have become downregulated from the improved transport capacity of amino acids.

Although other leucine transporters exist (e.g., LAT4), LAT1 is suggested to be most important for leucine transport, and it is essential for LAT1 to reside on the cellular membrane to fulfill transport (46, 47). This study extends the findings of Hodson et al. (43) who optimized a procedure for LAT1 IHC, and found LAT1 protein was greater in Type II muscle fibers. The present investigation found total LAT1 protein per fiber increased equally in all groups with training, which partly agrees with the western blot data. Total LAT1 protein was unaltered following training, which is likely due to the increased fCSA and less countable fibers in the field of view as well as the sensitivity differences between IHC and western blotting. Interestingly, we did not find LAT1 protein to increase along the sarcolemma following training. This notion suggests LAT1 may be internalized within the cell and translocated to the sarcolemma acutely in response to an anabolic stimulus similar to how LAT1 can be recruited to the lysosomal membrane (48). Moreover, Hyde et al. suggests internalization of SNAT2 with ceramide treatment (49) which suggests amino acid transporters have altered subcellular distribution following a given stimulus. Under basal conditions, LAT1 may be internalized and shuttled to the sarcolemma in response to resistance training or amino acid ingestion, although this is speculation.

To better understand if any of the molecular targets were related to changes in muscle fCSA, a simple linear regression analysis was performed. Following a correlation analysis, it was determined the change in ATF4 protein, albeit trending significance, was the only plausible candidate from measured markers to predict the change in muscle fCSA. Intriguingly, a relationship existed for LEU, but not PLA and WPC, and the significant regression equations were positive suggesting an increase in ATF4 protein was associated with skeletal muscle fiber hypertrophy. These findings are in direct conflict with previous literature suggesting ATF4 promotes muscle atrophy (22–24); however, it is paramount to note these findings are only respective to the changes seen following 12 weeks of resistance exercise training and do not reflect acute alterations and the ensuing responses that could have occurred during training. Importantly, the downregulation found in ATF4 protein following resistance exercise training supports that an attenuation in ATF4 protein promotes skeletal muscle hypertrophy, but the regression analysis suggests that acute alterations in ATF4 protein may be more important for determining skeletal muscle hypertrophy seen with exercise training. As noted, the change in ATF4 protein only predicted the change in muscle fCSA in the LEU group suggesting a unique relationship may exist between ATF4 protein and leucine supplementation. Moreover, it is possible the absence of the other BCAAs or amino acids in the presence of high leucine concentrations is causing this effect. Future investigations are needed to determine the precise relationship of ATF4 and leucine as well as other amino acids.

Amino acid metabolism, and specifically BCAA metabolism, is foundational for anaplerotic reactions. These reactions serve to meet metabolic demands while indirectly stimulating protein synthesis by limiting oxidation of amino acids (e.g., leucine). In spite of the fact that BCAT2 protein levels have been shown to be involved with metabolism and endurance performance (50, 51), our results suggest resistance exercise training or different supplementation paradigms do not impact this marker. The BCKDH complex is critical for BCAA metabolism given that it is the rate-limiting enzyme and is the commitment step toward generation of ATP from BCAA catabolism. BCKDHα protein levels increased with training herein, where a significantly greater increase occurred in the PLA vs. the LEU and WPC groups. The alpha-subunit of the complex is critical as phosphorylation of this subunit by the BCKDH kinase inactivates the complex (25). Unfortunately, due to limited resources and tissue, this investigation was unable to explore the protein content of other subunits of the BCKDH complex or associated regulatory enzymes such as the BCKDH kinase. Similarly, activity and phosphorylation of the BCKDH complex was also not measured, and these are limitations herein and warrant further investigation.

Given that resistance training was found to increase skeletal muscle LAT1 protein levels, *in vitro* experiments were performed to determine if LAT1 overexpression affected various molecular outcomes associated with anabolism (e.g., muscle protein synthesis and proteasome activity). While LAT1-tranfected cells possessed more LAT1 protein, intracellular amino acid concentrations (and leucine in particular) were not altered in LAT1-transfected vs. control cells. As mentioned above, this may be related to the lack of an anabolic stimulus required to localize LAT1 to the sarcolemma. Notwithstanding, a provocative and unexpected finding was that the LAT1-trasfected cells experienced an attenuation in puromycin incorporation suggesting rates of protein synthesis were lower. Notably, our laboratory has also observed that when LAT1 protein was knocked down in C2C12 myotubes, there was an increase in protein synthesis [(52); *unpublished observations*]. These findings are puzzling given the presumption that increased LAT1 protein would lead to increased intracellular leucine and, thus, increased protein synthesis. Interestingly, amino acid concentration nor proteasome activity was different between control and overexpression cells, but BCKDHα protein was lower in LAT1 overexpressed cells compared to control. The findings from cell culture in conjunction with the findings from humans regarding the different alterations in the BCKDHα protein suggests the BCKDH complex is not only important for metabolism but also affects protein synthesis. Indeed, dysregulation of the BCKDH complex results in maple syrup urine disease and is also associated with skeletal muscle atrophy (53, 54). Based on these findings, cellular transport of amino acids appears secondary to amino acid metabolism or, perhaps, overall metabolism given high glucose concentrations can limit amino acid oxidation (55). In attempt to reconcile the findings in this investigation in concert with findings from previous studies, increased LAT1 protein may promote greater efflux of amino acids from the sarcoplasm effectively lowering intracellular concentration and, perhaps, protein synthesis (48, 56). It is also possible the production of the BCKDH complex subunits turnover more slowly than transport proteins leading to a buildup of alpha-ketoacids which are toxic in excess. It is important to note these hypotheses are speculative and underpin the need for more research in this area.

In conclusion, the current investigation demonstrates total-body resistance exercise over 12 weeks affects amino acid metabolism and transporter content. While acute exercise was known to affect amino acid transporters, these data provide context to long-term adaptations in amino acid transporters in response to chronic resistance exercise. Indeed, resistance exercise training increases skeletal muscle LAT1, PAT1, and BCKDHα protein and decreases ATF4 protein. These markers are also affected by nutritional supplementation during training as shown by PLA participants experiencing the most pronounced alterations in these markers. The alterations in measured proteins do not appear to be associated with regulating skeletal muscle hypertrophy; however, regression analysis suggests a unique relationship between ATF4 and leucine. Lastly, overexpression of LAT1 protein decreased BCKDHα protein and protein synthesis without altering amino acid concentrations or proteasome activity, which suggests enzymes related to amino acid metabolism may affect protein synthesis.

## Data Availability Statement

The raw data supporting the conclusions of this article will be made available by the authors, without undue reservation.

## Ethics Statement

The studies involving human participants were reviewed and approved by Auburn University IRB. The patients/participants provided their written informed consent to participate in this study.

## Author Contributions

PAR, MDR, and CM conceived and designed research. PAR analyzed data and prepared figures. PAR and MDR interpreted results of experiments and drafted manuscript. All authors edited and revised manuscript, approved final version of manuscript, and assisted with data collection and performed experiments.

## Conflict of Interest

Funding for participant compensation and reagents were provided by monetary donations to MDR through Hilmar Ingredients (Hilmar, CA, USA), Bionutritional Research Group (Irvine, CA, USA), and Lockwood, LLC (Belton, TX, USA). Funding for the data procured in the current manuscript as well as article publishing charges were provided by a discretionary laboratory fund of MDR. Dr. Christopher Lockwood (Lockwood, LLC) was involved in the study design for the parent paper of this current manuscript (39). However, he had no involvement in the collection, analysis, interpretation of data, the writing of this current article or the decision to submit it for publication. The results of this study are presented clearly, honestly, and without fabrication, falsification, or inappropriate data manipulation. The laboratory of MDR performs contracted research for nutritional supplement companies. Moreover, MDR has served as a paid consultant with various nutritional supplement companies. However, MDR has not served as a consultant for Hilmar Ingredients, Bionutritional Research Group, or Lockwood, LLC. The remaining authors declare that the research was conducted in the absence of any commercial or financial relationships that could be construed as a potential conflict of interest.

## References

[1] Bodine SC. Disuse-induced muscle wasting. Int J Biochem Cell Biol. (2013) 45:2200–8. 10.1016/j.biocel.2013.06.011 23800384 PMC3856924

[2] Roberson PA Shimkus KL Welles JE Xu D Whitsell AL Kimball EM. A time course for markers of protein synthesis and degradation with hindlimb unloading and the accompanying anabolic resistance to refeeding. J Appl Physiol. (2020) 129:36–46. 10.1152/japplphysiol.00155.2020 32407240 PMC7469230

[3] Roberson PA Romero MA Mumford PW Osburn SC Haun CT Vann CG. Protein supplementation throughout 10 weeks of progressive run training is not beneficial for time trial improvement. Front Nutr. (2018) 5:97. 10.3389/fnut.2018.00097 30456213 PMC6230989

[4] Vann CG Osburn SC Mumford PW Roberson PA Fox CD Sexton CL. Skeletal muscle protein composition adaptations to 10 weeks of high-load resistance training in previously-trained males. Front Physiol. (2020) 11:259. 10.3389/fphys.2020.00259 32292355 PMC7135893

[5] Phillips SM Van Loon LJ. Dietary protein for athletes: from requirements to optimum adaptation. J Sports Sci. (2011) 29(Suppl. 1):S29–38. 10.1080/02640414.2011.619204 22150425

[6] Kato H Suzuki K Bannai M Moore DR. Protein requirements are elevated in endurance athletes after exercise as determined by the indicator amino acid oxidation method. PLoS ONE. (2016) 11:e0157406. 10.1371/journal.pone.0157406 27322029 PMC4913918

[7] Morton RW Murphy KT Mckellar SR Schoenfeld BJ Henselmans M Helms E. A systematic review, meta-analysis and meta-regression of the effect of protein supplementation on resistance training-induced gains in muscle mass and strength in healthy adults. Br J Sports Med. (2018) 52:376–84. 10.1136/bjsports-2017-097608 28698222 PMC5867436

[8] Volpi E Kobayashi H Sheffield-Moore M Mittendorfer B Wolfe RR. Essential amino acids are primarily responsible for the amino acid stimulation of muscle protein anabolism in healthy elderly adults. Am J Clin Nutr. (2003) 78:250–8. 10.1093/ajcn/78.2.250 12885705 PMC3192452

[9] Koopman R Wagenmakers AJ Manders RJ Zorenc AH Senden JM Gorselink M. Combined ingestion of protein and free leucine with carbohydrate increases postexercise muscle protein synthesis *in vivo* in male subjects. Am J Physiol Endocrinol Metab. (2005) 288:E645–53. 10.1152/ajpendo.00413.2004 15562251

[10] Koopman R Verdijk L Manders RJ Gijsen AP Gorselink M Pijpers E. Co-ingestion of protein and leucine stimulates muscle protein synthesis rates to the same extent in young and elderly lean men. Am J Clin Nutr. (2006) 84:623–32. 10.1093/ajcn/84.3.623 16960178

[11] Norton LE Wilson GJ Layman DK Moulton CJ Garlick PJ. Leucine content of dietary proteins is a determinant of postprandial skeletal muscle protein synthesis in adult rats. Nutr Metab. (2012) 9:67. 10.1186/1743-7075-9-67 22818257 PMC3488566

[12] Atherton PJ Smith K Etheridge T Rankin D Rennie MJ. Distinct anabolic signalling responses to amino acids in C2C12 skeletal muscle cells. Amino Acids. (2010) 38:1533–9. 10.1007/s00726-009-0377-x 19882215

[13] Wilkinson DJ Hossain T Hill DS Phillips BE Crossland H Williams J. Effects of leucine and its metabolite β-hydroxy-β-methylbutyrate on human skeletal muscle protein metabolism. J Physiol. (2013) 591:2911–23. 10.1113/jphysiol.2013.253203 23551944 PMC3690694

[14] Xu D Shimkus KL Lacko HA Kutzler L Jefferson LS Kimball SR. Evidence for a role for sestrin1 in mediating leucine-induced activation of mTORC1 in skeletal muscle. Am J Physiol Endocrinol Metab. (2019) 316:E817–28. 10.1152/ajpendo.00522.2018 30835510 PMC6580170

[15] Ögmundsdóttir MH Heublein S Kazi S Reynolds B Visvalingam SM Shaw MK. Proton-assisted amino acid transporter PAT1 complexes with Rag GTPases and activates TORC1 on late endosomal and lysosomal membranes. PLoS ONE. (2012) 7:e36616. 10.1371/journal.pone.0036616 22574197 PMC3344915

[16] Malmberg SE Adams CM. Insulin signaling and the general amino acid control response. Two distinct pathways to amino acid synthesis and uptake. J Biol Chem. (2008) 283:19229–34. 10.1074/jbc.M801331200 18480057

[17] Kilberg MS Shan J Su N. ATF4-dependent transcription mediates signaling of amino acid limitation. Trends Endocrinol Metab. (2009) 20:436–43. 10.1016/j.tem.2009.05.008 19800252 PMC3587693

[18] Drummond MJ Glynn EL Fry CS Timmerman KL Volpi E Rasmussen BB. An increase in essential amino acid availability upregulates amino acid transporter expression in human skeletal muscle. Am J Physiol Endocrinol Metab. (2010) 298:E1011–18. 10.1152/ajpendo.00690.2009 20304764 PMC2867366

[19] Drummond MJ Fry CS Glynn EL Timmerman KL Dickinson JM Walker DK. Skeletal muscle amino acid transporter expression is increased in young and older adults following resistance exercise. J Appl Physiol. (2011) 111:135–42. 10.1152/japplphysiol.01408.2010 21527663 PMC3137547

[20] Drummond MJ Dickinson JM Fry CS Walker DK Gundermann DM Reidy PT. Bed rest impairs skeletal muscle amino acid transporter expression, mTORC1 signaling, and protein synthesis in response to essential amino acids in older adults. Am J Physiol Endocrinol Metab. (2012) 302:E1113–22. 10.1152/ajpendo.00603.2011 22338078 PMC3361979

[21] Reidy PT Walker DK Dickinson JM Gundermann DM Drummond MJ Timmerman KL. Soy-dairy protein blend and whey protein ingestion after resistance exercise increases amino acid transport and transporter expression in human skeletal muscle. J Appl Physiol. (2014) 116:1353–64. 10.1152/japplphysiol.01093.2013 24699854 PMC4044402

[22] Ebert SM Monteys AM Fox DK Bongers KS Shields BE Malmberg SE. The transcription factor ATF4 promotes skeletal myofiber atrophy during fasting. Mol Endocrinol. (2010) 24:790–9. 10.1210/me.2009-0345 20197309 PMC2852358

[23] Ebert SM Dyle MC Bullard SA Dierdorff JM Murry DJ Fox DK. Identification and small molecule inhibition of an activating transcription factor 4 (ATF4)-dependent pathway to age-related skeletal muscle weakness and atrophy. J Biol Chem. (2015) 290:25497–511. 10.1074/jbc.M115.681445 26338703 PMC4646196

[24] Moro T Ebert SM Adams CM Rasmussen BB. Amino acid sensing in skeletal muscle. Trends Endocrinol Metab. (2016) 27:796–806. 10.1016/j.tem.2016.06.010 27444066 PMC5075248

[25] Shimomura Y Obayashi M Murakami T Harris RA. Regulation of branched-chain amino acid catabolism: nutritional and hormonal regulation of activity and expression of the branched-chain alpha-keto acid dehydrogenase kinase. Curr Opin Clin Nutr Metab Care. (2001) 4:419–23. 10.1097/00075197-200109000-00013 11568504

[26] Fujii H Shimomura Y Murakami T Nakai N Sato T Suzuki M Branched-chain alpha-keto acid dehydrogenase kinase content in rat skeletal muscle is decreased by endurance training. Biochem Mol Biol Int. (1998) 44:1211–6. 10.1080/15216549800202302 9623776

[27] Howarth KR Burgomaster KA Phillips SM Gibala MJ. Exercise training increases branched-chain oxoacid dehydrogenase kinase content in human skeletal muscle. Am J Physiol Regul Integr Comp Physiol. (2007) 293:R1335–41. 10.1152/ajpregu.00115.2007 17581840

[28] Mckenzie S Phillips SM Carter SL Lowther S Gibala MJ Tarnopolsky MA. Endurance exercise training attenuates leucine oxidation and BCOAD activation during exercise in humans. Am J Physiol Endocrinol Metab. (2000) 278:E580–7. 10.1152/ajpendo.2000.278.4.E580 10751189

[29] Shimomura Y Murakami T Nakai N Nagasaki M Harris RA Exercise promotes BCAA catabolism: effects of BCAA supplementation on skeletal muscle during exercise. J Nutr. (2004) 134:1583S−7S. 10.1093/jn/134.6.1583S 15173434

[30] Biolo G Maggi SP Williams BD Tipton KD Wolfe RR. Increased rates of muscle protein turnover and amino acid transport after resistance exercise in humans. Am J Physiol. (1995) 268:E514–20. 10.1152/ajpendo.1995.268.3.E514 7900797

[31] Trappe S Williamson D Godard M Porter D Rowden G Costill D. Effect of resistance training on single muscle fiber contractile function in older men. J Appl Physiol. (2000) 89:143–52. 10.1152/jappl.2000.89.1.143 10904046

[32] Roth SM Ivey FM Martel GF Lemmer JT Hurlbut DE Siegel EL. Muscle size responses to strength training in young and older men and women. J Am Geriatr Soc. (2001) 49:1428–33. 10.1046/j.1532-5415.2001.4911233.x 11890579

[33] Trappe S Godard M Gallagher P Carroll C Rowden G Porter D. Resistance training improves single muscle fiber contractile function in older women. Am J Physiol Cell Physiol. (2001) 281:C398–406. 10.1152/ajpcell.2001.281.2.C398 11443039

[34] Kosek DJ Kim JS Petrella JK Cross JM Bamman MM. Efficacy of 3 days/wk resistance training on myofiber hypertrophy and myogenic mechanisms in young vs. older adults. J Appl Physiol. (2006) 101:531–44. 10.1152/japplphysiol.01474.2005 16614355

[35] Dreyer HC Drummond MJ Pennings B Fujita S Glynn EL Chinkes DL. Leucine-enriched essential amino acid and carbohydrate ingestion following resistance exercise enhances mTOR signaling and protein synthesis in human muscle. Am J Physiol Endocrinol Metab. (2008) 294:E392–400. 10.1152/ajpendo.00582.2007 18056791 PMC2706121

[36] Moore DR Tang JE Burd NA Rerecich T Tarnopolsky MA Phillips SM. Differential stimulation of myofibrillar and sarcoplasmic protein synthesis with protein ingestion at rest and after resistance exercise. J Physiol. (2009) 587:897–904. 10.1113/jphysiol.2008.164087 19124543 PMC2669978

[37] Roberson PA Haun CT Mobley CB Romero MA Mumford PW Martin JS Skeletal muscle amino acid transporter and BCAT2 expression prior to and following interval running or resistance exercise in mode-specific trained males. Amino Acids. (2018) 50:961–5. 10.1007/s00726-018-2570-2 29725856

[38] Dickinson JM Gundermann DM Walker DK Reidy PT Borack MS Drummond MJ. Leucine-enriched amino acid ingestion after resistance exercise prolongs myofibrillar protein synthesis and amino acid transporter expression in older men. J Nutr. (2014) 144:1694–702. 10.3945/jn.114.198671 25332468 PMC4195415

[39] Mobley CB Haun CT Roberson PA Mumford PW Romero MA Kephart WC. Effects of whey, soy or leucine supplementation with 12 weeks of resistance training on strength, body composition, and skeletal muscle and adipose tissue histological attributes in college-aged males. Nutrients. (2017) 9:972. 10.3390/nu9090972 28869573 PMC5622732

[40] Romero MA Mobley CB Mumford PW Roberson PA Haun CT Kephart WC. Acute and chronic resistance training downregulates select LINE-1 retrotransposon activity markers in human skeletal muscle. Am J Physiol Cell Physiol. (2018) 314:C379–88. 10.1152/ajpcell.00192.2017 29351416

[41] Roberson PA Romero MA Osburn SC Mumford PW Vann CG Fox CD. Skeletal muscle LINE-1 ORF1 mRNA is higher in older humans but decreases with endurance exercise and is negatively associated with higher physical activity. J Appl Physiol. (2019) 127:895–904. 10.1152/japplphysiol.00352.2019 31369326

[42] Roberts MD Mobley CB Vann CG Haun CT Schoenfeld BJ Young KC. Synergist ablation-induced hypertrophy occurs more rapidly in the plantaris than soleus muscle in rats due to different molecular mechanisms. Am J Physiol Regul Integr Comp Physiol. (2019) 318:R360–8. 10.1152/ajpregu.00304.2019 31850817

[43] Hodson N Brown T Joanisse S Aguirre N West DWD Moore DR. Characterisation of L-type amino acid transporter 1 (LAT1) expression in human skeletal muscle by immunofluorescent microscopy. Nutrients. (2017) 10:23. 10.3390/nu10010023 29278358 PMC5793251

[44] Carpenter AE Jones TR Lamprecht MR Clarke C Kang IH Friman O. CellProfiler: image analysis software for identifying and quantifying cell phenotypes. Genome Biol. (2006) 7:R100. 10.1186/gb-2006-7-10-r100 17076895 PMC1794559

[45] Stokes T Hector AJ Morton RW Mcglory C Phillips SM. Recent perspectives regarding the role of dietary protein for the promotion of muscle hypertrophy with resistance exercise training. Nutrients. (2018) 10:180. 10.3390/nu10020180 29414855 PMC5852756

[46] Yanagida O Kanai Y Chairoungdua A Kim DK Segawa H Nii T. Human L-type amino acid transporter 1 (LAT1): characterization of function and expression in tumor cell lines. Biochim Biophys Acta. (2001) 1514:291–302. 10.1016/S0005-2736(01)00384-4 11557028

[47] Kim CH Park KJ Park JR Kanai Y Endou H Park JC. The RNA interference of amino acid transporter LAT1 inhibits the growth of KB human oral cancer cells. Anticancer Res. (2006) 26:2943–8. 16886618

[48] Milkereit R Persaud A Vanoaica L Guetg A Verrey F Rotin D. LAPTM4b recruits the LAT1-4F2hc Leu transporter to lysosomes and promotes mTORC1 activation. Nat Commun. (2015) 6:7250. 10.1038/ncomms8250 25998567 PMC4455107

[49] Hyde R Hajduch E Powell DJ Taylor PM Hundal HS. Ceramide down-regulates system A amino acid transport and protein synthesis in rat skeletal muscle cells. FASEB J. (2005) 19:461–3. 10.1096/fj.04-2284fje 15611152

[50] She P Zhou Y Zhang Z Griffin K Gowda K Lynch CJ. Disruption of BCAA metabolism in mice impairs exercise metabolism and endurance. J Appl Physiol. (2010) 108:941–9. 10.1152/japplphysiol.01248.2009 20133434 PMC2853195

[51] Lynch CJ Kimball SR Xu Y Salzberg AC Kawasawa YI. Global deletion of BCATm increases expression of skeletal muscle genes associated with protein turnover. Physiol Genomics. (2015) 47:569–80. 10.1152/physiolgenomics.00055.2015 26351290 PMC4629004

[52] Mobley CB Mumford PW Mccarthy JJ Miller ME Young KC Martin JS. Whey protein-derived exosomes increase protein synthesis and hypertrophy in C2C12 myotubes. J Dairy Sci. (2017) 100:48–64. 10.3168/jds.2016-11341 28341051

[53] Nobukuni Y Mitsubuchi H Akaboshi I Indo Y Endo F Yoshioka A. Maple syrup urine disease. Complete defect of the E1 beta subunit of the branched chain alpha-ketoacid dehydrogenase complex due to a deletion of an 11-bp repeat sequence which encodes a mitochondrial targeting leader peptide in a family with the disease. J Clin Invest. (1991) 87:1862–6. 10.1172/JCI115209 2022752 PMC295312

[54] Sonnet DS O'leary MN Gutierrez MA Nguyen SM Mateen S Hsu Y Metformin inhibits branched chain amino acid (BCAA) derived ketoacidosis and promotes metabolic homeostasis in MSUD. Sci Rep. (2016) 6:28775 10.1038/srep28775 27373929 PMC4931503

[55] Shao D Villet O Zhang Z Choi SW Yan J Ritterhoff J. Glucose promotes cell growth by suppressing branched-chain amino acid degradation. Nat Commun. (2018) 9:2935. 10.1038/s41467-018-05362-7 30050148 PMC6062555

[56] Scalise M Galluccio M Console L Pochini L Indiveri C. The human SLC7A5 (LAT1): the intriguing histidine/large neutral amino acid transporter and its relevance to human health. Front Chem. (2018) 6:243. 10.3389/fchem.2018.00243 29988369 PMC6023973

