# Supplementary material for: LAT1 Protein Content Increases Following 12 Weeks of Resistance Exercise Training in Human Skeletal Muscle
Source: Front Nutr. 2021 Jan 14;7:628405. doi: 10.3389/fnut.2020.628405 (PMC7840583; doi:10.3389/fnut.2020.628405)
Supplement: Supplementary file 1 [file Image_1.pdf]

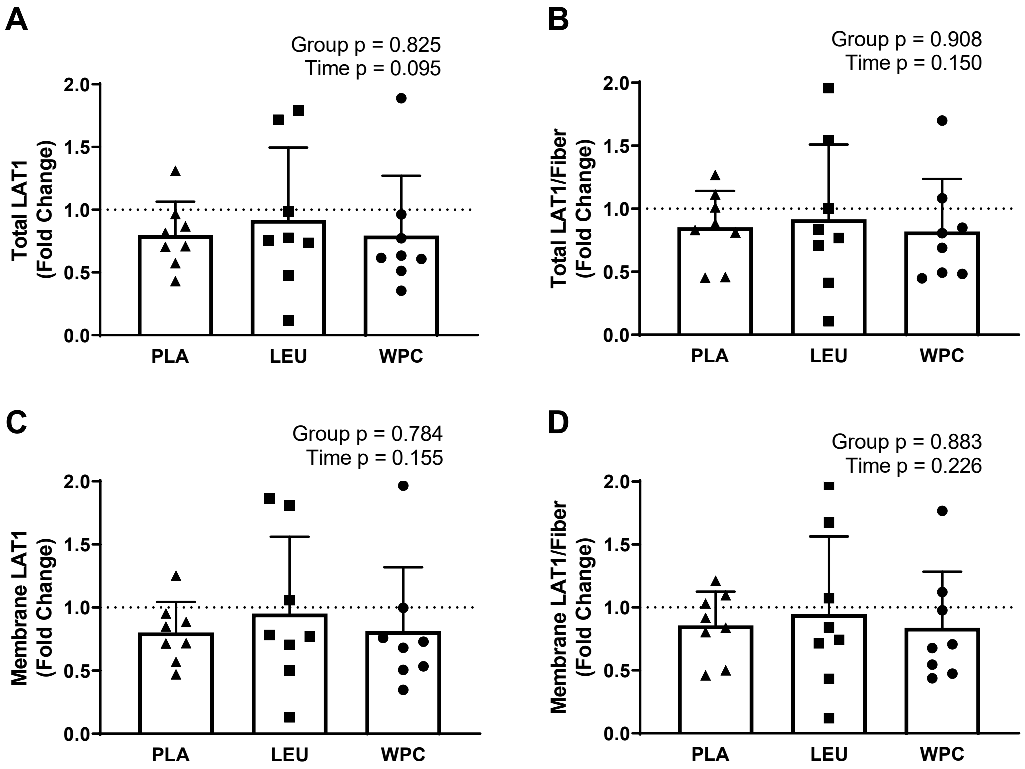

Figure S1. LAT1 protein is unaltered when using the antibody not designated for immunohistochemistry and no metric correlated with the antibody designated for immunohistochemistry. The fold change from PRE, designated as the dashed line at 1.00, for Total LAT1 (A), Total LAT1 per fiber (B), Membrane LAT1 (C), and Membrane LAT1 per fiber (D) protein content for each group using immunohistochemistry. Data are represented as mean ± standard deviation. Sample size for each group is 8.
